# Impact of the COVID-19 Pandemic on Patients With Acute Coronary Syndrome: A Tertiary Center Experience With Primary Percutaneous Intervention and Early Invasive Strategy

**DOI:** 10.7759/cureus.20747

**Published:** 2021-12-27

**Authors:** Wesam Alhejily

**Affiliations:** 1 Cardiology, King Abdulaziz University Faculty of Medicine, Jeddah, SAU; 2 Medicine, Dr. Sulaiman Al Habib Medical Group, Riyadh, SAU

**Keywords:** pandemic, outcomes, time, myocardial infarction, covid-19

## Abstract

Objective: This study aimed to determine the impact of the coronavirus disease 2019 (COVID-19) pandemic on patients with acute coronary syndrome (ACS).

Methods: This retrospective longitudinal cohort study analysed ACS patients admitted in a large tertiary center in 2019 (pre-pandemic) and 2020 (pandemic). The primary endpoint was mortality from myocardial infarction; secondary endpoints were death from any causes, including COVID-related complications, stroke, and coronary artery bypass graft surgery.

Results: There were 489 ACS patients admitted in 2020, and 614 in 2019, representing a 21% reduction (p=0.001). Male patients comprised 73% of the patients. Only eight were polymerase chain reaction (PCR)-confirmed positive COVID-19 patients. The mean time to presentation from the time of onset of symptoms in acute ST-elevation myocardial infarction cases was 48±16 in 2020 (vs. 4±3 h in 2019); this significant delay was observed in more than 50% of patients (p=0.0001). Mortality due to ACS in 2020 doubled, with eight patients confirmed dead during or within 30 days of admission, with none of the deaths related to COVID-19. The incidence of stroke (p=0.01) and coronary artery (p=0.0001) bypass was also high in 2020.

Conclusion: We found a statistically significant increase in the mortality related to myocardial infarction. Despite timely interventions, patients presented late and were worse than in the non-pandemic period.

## Introduction

The novel coronavirus disease 2019 (COVID-19), which can lead to acute respiratory distress syndrome, was first reported in December 2019 in Wuhan, China. Owing to its vast and rapid spread across the globe, the World Health Organization declared this global health emergency a pandemic on March 11, 2020 [1]. By December 2020, more than 80 million COVID-19 cases were reported, accounting for approximately two million direct deaths [2,3]. The number of acute coronary syndrome (ACS) cases typically increases during the flu season due to an increase in inflammatory markers leading to atherosclerotic plaque instability, which was confirmed in the autopsy of patients dying from common flu [4,5].

In a survey conducted by Pessoa-Amorim et al. across many hospitals worldwide, 78.8% of the participants responded that the number of hospital admissions of patients presenting with acute ST-elevation myocardial infarction (STEMI) decreased by more than 40% after the coronavirus outbreak [6]. Most participants reported that STEMI patients presented later than usual, and that >40% of STEMI patients admitted to hospital presented beyond the optimal window for primary percutaneous intervention (PCI) or thrombolysis. Predictors of delayed STEMI presentation included a country in total lockdown, >100 COVID-19 cases admitted locally, and the complete restructuring of the local cardiology service.^ ^Any delay in treatment related to acute ST-elevation myocardial infarction (MI) affects the patients’ life and outcomes, particularly in those severely ill with cardiogenic shock. ​​​​​​Scholz et al. found that every 10-minute delay in treatment resulted in 3.31 additional deaths per 100 PCI-treated patients [7].

Saudi Arabia has enforced several strict measures to contain the spread of the infection. For example, the seasonal Umrah and Pilgrimage for local and international worshippers were suspended. Malls and public places, such as parks, mosques, restaurants, and cafes, were closed, and classes were suspended. A shift to work from home was also adopted. In addition, a complete lockdown and partial and complete curfew were imposed several times to halt the spread of the virus. International and domestic travel was also restricted. These measures led to greater containment and control of the virus in Saudi Arabia compared with other countries where the number of infections has already spiraled out of control. The first confirmed case of COVID-19 infection in Saudi Arabia was reported on March 22, 2020. By December 31, the total number of cases reached more than 300,000, and about 6000 deaths were reported [8]. Public awareness campaigns were amplified on Ministry of Health (MOH) websites and different social media platforms. Daily press conferences with updates on the number of new confirmed cases and deaths were conducted, highlighting the possible causes of some case clusters. Patients from different backgrounds were guided using a toll-free hotline of the MOH. Regarding the healthcare setting, elective surgical operations were put on hold, appointments were rescheduled, and hospitals were mainly dedicated to treating emergency cases and patients with complicated COVID-19. Many field hospitals were also constructed in anticipation of patient overflow.

Despite the stress on the timely presentation of MI, we noticed a dramatic decline in the number of MI cases, particularly when curfew was in effect. Thus, in this prospective cohort study, we analysed the data of patients with ACS who were diagnosed during the pandemic, namely 2020, and compared their outcomes with those diagnosed in 2019. We aimed to assess the indirect impact of the pandemic on ACS patients.

## Materials and methods

The characteristics and clinical data of all patients with ACS during the COVID-19 pandemic were collected and compared with those from the same months of the previous year; data were gathered using the health information system database repository, in addition to catheterization laboratories databank in a large tertiary hospital with primary PCI capabilities. Mortality and surrogate endpoints, such as time from the onset of symptoms to presentation, door-to-balloon time and ejection fraction, were analysed. The primary endpoint was mortality related directly to ACS. The secondary endpoint was the composite of death from any cause, including COVID-19 infection, recurrent MI, or heart failure within 30 days. Analysis of variance (ANOVA) was used to examine the differences in variance across the means. Two-tailed tests were performed, and statistical significance was set at p = 0.05. Patient characteristics and risk factors were compared. Other parameters, such as the need for emergency coronary artery bypass graft (CABG) surgery and the incidence of acute stroke, were also studied. Results were tabulated and graphed for comparison; data, including age and door-to-balloon time, are presented as means ± standard deviations. The study protocol was approved by the institutional review board research committee, with access to the database. All patient-related information was anonymized and was Health Insurance Portability and Accountability Act compliant. ANOVA was used as we had categorical independent variables like death, stroke and CABG (with two or more categories); a paired t-test was used for continuous variables like time. All computations were made using IBM SPSS Statistics, version 22 (IBM Corp., Armonk, NY).

## Results

**Figure 1:**
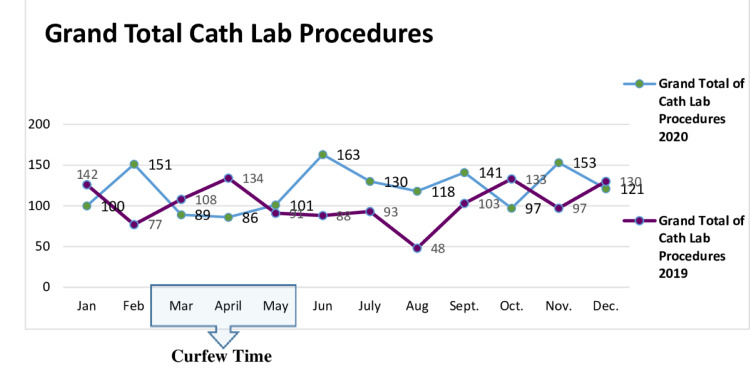
Monthly number of catheterization laboratory procedures in 2019 (violet line) and 2020 (blue line). Months when curfew was implemented are enclosed in a rectangle.

This retrospective cohort analysis of patients with ACS was conducted from January 2020 to December 31, 2020. We noted a decline of 17% in cardiac catheterization laboratories cases during the curfew time from early March to mid-May 2020 (Figure 1).

We identified 489 ACS cases in 2020 and 614 cases in the year prior to the pandemic. All patients were admitted to hospitals, with a 21% reduction in the number of admissions. Of these, 357 patients were male, representing 73% of the population. The dominant risk factors were hypertension (53%), diabetes (51%), and hypercholesteremia (5%). Risk factors were comparable (Table 1).

**Table 1:** Characteristics of patients with ACS ACS, acute coronary syndrome; COVID-19, coronavirus disease 2019; CABG, coronary artery bypass graft *Statistically significant p<0.05

ACS patients’ characteristics	2019 (pre-COVID-19)	2020 (during COVID-19)	p value*
Total number	N=614	N=489	0.001*
Mean age	53±7	55±7	0.4
Gender (male/female)	319/295	357/132	0.15
Nationality (Saudi/non-Saudi)	465/149	376/113	0.59
Hypertension	235	260	0.8
Diabetes	217	249	0.7
Smoking	70	30	0.046*
Need for CABG	9	26	0.032
Atrial fibrillation	14	12	0.06
Renal Impairment	56	69	0.08
Hyperlipidemia	57	24	0.05
Mean ejection fraction	50±5	48±5	0.9
Time to presentation	4 ± 3 hours	48 ± 16 hours	0.0001*

Only eight cases of reverse transcriptase-polymerase chain reaction (RT-PCR)-confirmed COVID-19 infection were related to an acute coronary event, despite a total number of 6000 confirmed COVID-19 cases. There were six non-STEMI cases; the delay to catheterization was related to the time needed for result confirmation, which took 24 hours. Only two of these cases were STEMI and were taken to the catheterization laboratory without delay. Swabs were collected en route to the cardiac catheterization laboratory, and diagnosis was made after primary PCI was performed. Visual triage using a questionnaire was conducted for all those coming to the hospital, along with temperature measurement, and this may have contributed to some delays in starting cases in the catheterization laboratories. Patients identified as high risk were swabbed by trained staff in full personal protection equipment. As we applied the primary PCI strategy to all coming with STEMI and an early invasive strategy to all other ACS categories within 4-24 hours, a zero-infection rate among the catheterization laboratory team was reported.

The mean time to presentation from the onset of symptoms in STEMI cases was 48±16 hours compared to 4±3 hours with a significant delay during the COVID-19 pandemic in more than 50% of the patients (p=0.0001). However, the mean door-to-balloon time was 89.91 and 106.14 minutes for patients presenting directly and indirectly to the hospital; for patients who presented to other facilities, the time was calculated from the first medical contact to balloon deployment instead of time from door to balloon. Times were higher than the time of the last year of 76.54 and 102.31 minutes, but still within the 90- and 120-minute range, respectively This was mainly related to more strict measures related to traffics and triaging patients and medical staff attending hospitals. The mean ejection fraction was 48±5%, lower than the last year before the COVID-19 pandemic 50±5%. Mortality due to ACS doubled during the pandemic, with eight patients confirmed dead; seven of these cases were related to arrhythmogenic death at presentation and one was related to ruptured ventricular septum that was discovered after 14 days of hospital discharge, versus only four cases in the year earlier. This was despite the primary PCI policy being applied to all those coming, with no significant difference in door-to-balloon time for STEMI cases. Since there was no direct death related to COVID-19, all-cause mortality and death due to ACS were similar (Table 2).

**Table 2:** Classification of ACS patients admitted during COVID-19 and pre-COVID-19 STEMI, ST-elevation myocardial infarction; ACS, acute coronary syndrome; COVID-19, coronavirus disease 2019; NSTEMI, non-ST-elevation myocardial infarction *Statistically significant p<0.05

Classification	2019	2020	p value
STEMI	55 (8.9%)	42 (8.6%)	0.89
Mean door-to-balloon time (direct)	76.54 min	89.91 min	0.68
Mean first medical contact to balloon time	102.31 min	106.14 min	0.75
NSTEMI	16 (2.6%)	28 (5.7%)	0.046*
Unstable angina	543 (88%)	419 (86%)	0.78
Use of intra-aortic balloon pump	10 (1.6%)	1 (0.2%)	0.001*
Use of inotropic support	8 (1.3%)	2 (0.4%)	0.01*
COVID-19-positive status	0 (0%)	8 (1.6%)	0.04*

With regard to major adverse outcomes, the rate of re-infarction (defined by a new rise in biomarkers with clinical symptoms of ACS within 30 days of discharge) was insignificant between both years. There was a trend of a higher rate of heart failure, defined by the presence of congestive symptoms, with an elevated prohormone of brain natriuretic peptide (pro BNP) level and a reduced ejection fraction of 35% or less either on initial presentation (23 cases representing 80%) or on readmission within 30 days after discharge (6 cases representing 20%), and it was statistically insignificant. Cases were managed with optimal medical therapy following international guidelines, after initial diuresis. Patients were placed on maximally tolerated therapy of angiotensin receptor neprilysin inhibitors (ARNIs), beta blockers, aldosterone antagonists, and sodium-glucose co-transporter 2 (SGLT2) inhibitors in addition to anti-ischemic therapy and anticoagulation as needed. For patients with renal failure or hyperkalemia, hydralazine and long-acting nitrate were used to substitute ARNIs and aldosterone antagonists. In contrast, there was a major increase in the rate of stoke with four cases documented to have had stroke, three during admission and one after 30 days of initial diagnosis compared to none from the year before the pandemic (p=0.001); the need for urgent coronary artery bypass surgery (CABG) was higher in the COVID year, with 24 versus 9 cases with a threefold increase (p=0.001). Interestingly, there was more usage of intra-aortic balloon pump (IABP) in the year prior to the pandemic likely explained by the fact that those patients presented with features of pump failure were past the window of revascularization and that they were not hemodynamically compromised; on the other hand, most deaths related to ACS were before patients were physically present in the catheterization laboratory precluding any interventions (Table 3).

**Table 3:** Mortality, MACE, CABG and stroke in different ACS populations MACE, major adverse cardiac events; ACS, acute coronary syndrome; COVID-19, coronavirus disease 2019; CABG, coronary artery bypass graft *Significant p value <0.05

Parameters	Pre-COVID-19 (2019)	During COVID-19 (2020)	p value
All-cause mortality, n (%)	4 (0.6)	8 (1.7)	0.01*
MACE, n (%) (death, re-infarction and heart failure)	39 (6)	41 (8)	0.56
Death related to ACS	4 (all during initial admission)	8 (7 during initial admission and 1 within 30 days after discharge)	0.01*
Re-infarction within 30 days from initial ACS	3	4	0.1
Heart failure, n (%)	32 (5.2) (all during initial admission)	29 (5.9) (23 at admission, 6 within 30 days after discharge)	0.1
CABG, n	9	24	0.001*
Stroke, n	0	4	0.01*

## Discussion

This study addressed the difference in outcomes for ACS related to the COVID-19 pandemic in the Kingdom of Saudi Arabia. Saudi Arabia has vast experience in responding to communicable diseases, which was gained from dealing with the Middle East respiratory syndrome during Hajj (pilgrimage), where more than 2.5 million worshippers from all over the world gathered for five days [9,10].

In the first three months of curfew implementation, we noted a decline in ACS cases, and the total number of cases performed in the cardiac catheterization laboratories. We speculate that this is likely because of the fear of going to hospitals because of the high rate of COVID-19 cases. In our hospital, 6000 confirmed cases was reported during the first 12 months of the pandemic; more than 800 cases were admitted, and more than 50 cases of mortality were reported. When the curfew was lifted, there was an increase in the number of ACS patients, and more patients presented with ACS, including late-presentation STEMI cases (Figure 1). In addition, the number of total procedures performed decreased by 17%, and ACS cases presented to us decreased by 21% compared to the pre-pandemic year 2019. As noted in the previous section, the number of deaths in 2020 remained low at 1.6% (n=8). One patient was pronounced dead in the catheterization lab, whereas most patients died within 4-30 days, either during hospitalization or after discharge. The number of deaths in 2020 was significantly higher than that in 2019, which had a death rate of <0.6%. Re-infarction was defined as a second presentation with a new rise in troponin and a clinical picture of ACS; this was documented in three versus four cases in the pre-pandemic compared to the pandemic year. The impact of having early invasive strategy for all coming during the pandemic might have played a major role in reducing this number compared to previous series, which showed a higher rate of mortality reaching more than 20% [11-21]. The cases seen during the pandemic had more established mechanical complications like ruptured ventricular septum and acute mitral regurgitation, but still dominated by pump failure and poor ejection fraction; left main and more diffuse three-vessel coronary artery diseases were more prevalent as the rate of bypass surgery was higher. Although the mean ejection fraction was comparable between both groups, the number of cases of heart failure was numerically higher during the pandemic time, 5.9% versus 5.2%, respectively, but statistically insignificant; this was directly related to the delay from the time of onset of symptoms, which was over two days compared to five to six hours with other patients in the non-COVID period.

Regarding major adverse outcomes, we noted an increase in the overall rate of thromboembolic stroke cases coming to the hospital, particularly among ACS patients. We noted four patients with ACS who had stroke following their admission, which was likely a thromboembolic event and related to cardiac sources of embolism. Particularly, three patients had atrial fibrillation, left ventricular intramural thrombus due to poor ejection fraction, and one patient had no identifiable source of embolus. The door-to-balloon time for patients arriving directly to the emergency department or transferred from other hospitals was longer during the pandemic than during the pre-pandemic period, ranging from 90 to 120 minutes. We also noted a lower use of IABP and ionotropic support among ACS patients during the pandemic than during the pre-pandemic time. This is likely related to initial stable hemodynamics, or early death as most patients would have developed complications from MI prior to the admission period, leading to their demise and precluding any interventions.

This study had some limitations. First, this was a single-center study. Second, only a low prevalence of COVID-19 cases among ACS patients was observed. Despite these limitations, this retrospective analysis study provides valuable information on ACS patients during the COVID 19-pandemic. We assessed the indirect impact of the pandemic on such vulnerable patients, and the delay in their presentation that may have deleterious effects on their lives, thus highlighting the importance of health education of the public [22-30]. Public awareness campaigns highlighting the seriousness of these symptoms may play a role in ameliorating this effect. Future studies should be conducted to examine the long-term impact of the pandemic on the study population and the effect of public awareness campaigns on ACS-related symptoms and the need for timely presentation to healthcare facilities.

## Conclusions

This retrospective cohort study revealed the indirect effect of the COVID-19 pandemic on patients with ACS. We noted a higher number of deaths, complications, and the need for urgent CABG and stroke. Despite the early invasive strategy, mortality was related to a delay in presentation from the time of onset of symptoms.
